# ﻿New taxa and a combination in Glomerales (Glomeromycota, Glomeromycetes)

**DOI:** 10.3897/mycokeys.112.136158

**Published:** 2025-01-22

**Authors:** Janusz Błaszkowski, Szymon Zubek, Paweł Milczarski, Ryszard Malinowski, Piotr Niezgoda, Bruno Tomio Goto

**Affiliations:** 1 Department of Environmental Management, West Pomeranian University of Technology in Szczecin, Słowackiego 17, PL-71434 Szczecin, Poland; 2 Institute of Botany, Faculty of Biology, Jagiellonian University, 30-387, Kraków, Poland; 3 Department of Genetic, Plant Breeding & Biotechnology, West Pomeranian University of Technology in Szczecin, Słowackiego 17, PL-71434 Szczecin, Poland; 4 Department of Environmental Management, West Pomeranian University of Technology in Szczecin, Słowackiego 17, PL–71434 Szczecin, Poland; 5 Departamento de Botânica e Zoologia, Universidade Federal do Rio Grande do Norte, Campus Universitário, 59078–900, Natal, RN, Brazil

**Keywords:** Arbuscular mycorrhizal fungi, morphology, nuc rDNA, phylogenetic taxonomy, *rpb1*

## Abstract

This article presents the results of morphological studies, as well as comparisons and phylogenetic analyzes of sequences of four arbuscular mycorrhizal fungi (AMF, phylum Glomeromycota): *Dominikiaindica*, *Dominikiaindica* strain 211, Isolate 517, and Isolate 524. *Dominikiaindica* strain 211 was previously characterized only by sequences of the 45S nuc rDNA region (= 18S, partial, ITS-1-5.8S-ITS2, 28S, partial) and the *rpb1* gene (without any morphological data) that were deposited in GenBank under the incorrect name “*Dominikiaindica* strain 211”. Its 45S sequences differed from the original *D.indica* sequences and, consequently, resulted in erroneous phylogenetic classification of this species. Isolate 517 and Isolate 524 slightly differed in morphology from *Macrodominikiacompressa* (formerly *D.compressa*) and *Microkamienskiaperpusilla* (formerly *Kamienskiaperpusilla*), respectively. *Microkamienskiaperpusilla* was originally found in a maritime dune site of Italy in 2009 and not yet reported from any other habitat in the world. Our sequence comparisons and analyses showed that *D.indica* represents a new genus, here created under the name *Delicatispora***gen. nov.** with *De.indica***comb. nov.**, and *Dominikiaindica* strain 211 is a new species, described as *Dominikiaparaminuta***sp. nov.** These analyses also indicated that Isolate 517 is conspecific to *M.compressa* and confirmed the correctness of the transfer of *D.compressa* by other AMF researchers to *Macrodominikia* gen. nov. with *M.compressa* comb. nov. Morphological studies of our *M.compressa* specimens grown in culture showed that the original description of this species is incomplete and, therefore, the description was emended. Phylogenetic analyses of Isolate 524 proved its conspecificity to *Mk.perpusilla* and thus revealed its second site of occurrence, i.e., the coastal dunes of the Hel Peninsula in northern Poland.

## ﻿Introduction

The phylum Glomeromycota includes arbuscular mycorrhizal fungi living in symbiosis with ca. 70% vascular plants, mainly inhabiting terrestrial sites (Smith and Read 2008), rarely aquatic (Queiroz et al. 2020, 2022; Gomes et al. 2022). To date, the ca. 370 species of this phylum were distributed in three classes, six orders, 17 families, and 49 genera (Goto et al 2024; Wijayawardene et al. 2024).

The genera *Dominikia* and *Kamienskia*, considered in this study, were described in the family Glomeraceae sensu Piroz. & Dalpé of Glomeromycota following phylogenetic analyses of 45S nuc rDNA (= 18S-ITS-28S) sequences of five species originally described in the genus *Glomus*, the newly described *Dominikiadisticha*, and an isolate called *Dominikia* 211 (Błaszkowski et al. 2015a). The type species of *Dominikia* and *Kamienskia* were *D.minuta* and *K.bistrata*, respectively. The second species of *Kamienskia* was *K.perpusilla*.

*Dominikia* species produce glomoid spores sensu Morton and Redecker (2001) that arise blastically at tips of cylindrical or funnel-shaped sporogenous hyphae, like spores of *G.macrocarpum*, the type species of *Glomus*, Glomeraceae sensu Piroz. & Dalpé, and Glomeromycota (Schüßler and Walker 2010). Except for *D.difficilevidera* and *D.glomerocarpica*, which produce spores singly in the soil and in compact epigeous glomerocarps (= sporocarps) with a peridium, respectively (Błaszkowski et al. 2015b, 2021a), spores of the other species are formed in loose to compact hypogeous clusters without a peridium (Błaszkowski et al. 2000, 2009a, 2009b, 2010a, 2010b, 2015a, 2015b, 2016, 2021c, Oehl et al. 2003, 2014, 2015, Yu et al. 2022). Spores of *D.aurea*, *D.bernensis*, *D.compressa*, *D.duoreactiva*, and *D.gansuensis* are clearly darker colored (pale yellow to yellow brown) (Oehl et al. 2003, 2014, 2015; Błaszkowski 2012; Błaszkowski et al. 2015b, Yu et al. 2022) than those of the other species, of which six are colorless, and the others orange yellow at most (Błaszkowski 2000, 2009a, 2010a, 2010b, 2015a, 2016, 2021c). The spores of the vast majority of *Dominikia* species are very small (12–86 µm diam when globose); only those of *D.compressa* are slightly larger (50–103 µm diam) (Oehl et al. 2014).

To date, *Dominikia* comprised 14 species. However, the taxonomic statuses of two of them, i.e., *D.compressa* and *D.indica*, which were originally described as *G.compressum* and *G.indicum* (Błaszkowski et al. 2010b; Oehl et al. 2014), have been uncertain. Very recently, Silva et al. (2024) separated four new families from Glomeraceae and transferred *D.compressa* to a new genus, *Macrodominikia*, with *M.compressa* comb. nov. in Dominikiaceae fam. nov., making this species’ status clear. In addition, phylogenetic analyses, other than those mentioned above, of *Dominikia* species often included *Dominikia* 211. The morphological features of this isolate have not yet been made public, and its molecular sequences have caused taxonomic confusion described below.

As to *D.indica*, in the article describing this fungus as a new species in *Glomus* (Błaszkowski et al. 2010b), it was shown to be a phylogenetic sister of *G.achrum*, which was later renamed *D.achra* (Błaszkowski et al 2015b). Oehl’s et al. (2015) and Al-Yahya’ei’s et al. (2017) phylogenetic analyses accommodated *D.indica* in a sister position to *D.aurea*. In the Błaszkowski’s et al. (2015b) phylogenetic tree, *D.indica* was placed in a basal clade to the other *Dominikia* species, but the *Dominikia* clade obtained only Bayesian inference support (BI = 1.0), while the maximum likelihood (ML) support was insignificant (= 58%). Subsequent Błaszkowski’s et al. (2018b) analyses showed *D.indica* to be a sister to *D.litorea*.

Corazon-Guivin et al. (2019a) transferred *D.litorea* to the new genus *Microdominikia* with *M.litorea* comb. nov. In the same study and that by Silva et al. (2024), Bayesian inference and ML phylogenetic analyses of 45S sequences revealed *D.indica* as the sister of *D.bernensis*. Instead, Błaszkowski et al. (2021c) and Yu et al. (2022) showed *D.indica* to be a sister of *D.minuta*. However, the relationships found resulted from the use of the KJ564163, KJ564164, KJ564167, and KJ564169 sequences, which were erroneously ascribed in GenBank to *D.indica* under the name *Dominikiaindica* strain 211. *Dominikiaindica* was originally phylogenetically characterized based on the GU059544–GU059549 sequences (Błaszkowski et al. 2010b).

In the *Dominikia* phylogenies reconstructed by Błaszkowski et al. (2015a) and Błaszkowski et al. (2018c), *Dominikia* 211 was the sister group of *D.minuta* and *D.litorea* (= *M.litorea*), respectively.

Regarding *Kamienskiaperpusilla*, Corazon-Guivin et al. (2019b), based on phylogenetic analyses and high genetic differences, transferred this species to *Microkamienskia* gen. nov. with the type species *M.perpusilla*. Currently, *Microkamienskia* includes three species producing hyaline and very small (10–35 µm diam when globose) glomoid spores, which arise in loose to compact hypogeous clusters (Błaszkowski et al. 2009a, 2016; Corazon-Guivin et al. 2019b). Silva et al. (2024) placed *Microkamienskia* in Kamienskiaceae fam. nov.

The identity values and phylogenetic analyses of environmental sequences deposited in public databases suggest that the so far characterized *Dominikia* species and the other major taxa discussed here represent a small part of those functioning in various habitats around the world. The reasons for this poor understanding of the species diversity of this group of AMF have been discussed by Błaszkowski et al. (2010b, 2015b, 2018c, 2021a) and Corazon-Guivin et al. (2019a, 2019b).

We grew in culture the AM fungi called *Dominikia* 211, Isolate 517 and Isolate 524, which originated from maritime dunes of Poland. Preliminary comparisons of sequences and phylogenetic analyses suggested that Isolate 517 and Isolate 524 are conspecific to *M.compressa* and *Microkamienskiaperpusilla*, respectively, and *Dominikia* 211 is an undescribed species.

The aims of our subsequent studies were (i) to verify the phylogeny of *D.indica*, (ii) to describe and illustrate the morphology of *Dominikia* 211 and determine its phylogenetic status and position among sequenced members of Glomerales, (iii) to characterize the morphology and phylogeny of Isolate 517 and compare its characteristics with those of *M.compressa*, and (iv) to check whether Isolate 524 is conspecific to *Mk.perpusilla*.

## ﻿Materials and methods

### ﻿Origin of study material

Of the three main AMF analyzed here, only *Dominikia* 211 and Isolate 524 (numbers are from an AMF database maintained by J. Błaszkowski) were characterized based on spores extracted from single-species pot cultures. Isolate 517 came from a trap pot culture because numerous attempts to grow this fungus in single-species cultures failed. The single-species cultures were established from spores extracted from trap cultures. All trap cultures were inoculated with field mixtures of rhizosphere soil and root fragments of *Ammophilaarenaria* (L.) Link. that colonized maritime foredunes of the Hel Peninsula in northern Poland. *Ammophilaarenaria* was the only plant species occurring in the sampled sites. According to Sayre et al. (2020), the climate type on the Hel Peninsula is cool temperate moist. Mean temperature and rainfall are −1.1 °C and 38 mm in January and 17.1 °C and 67 mm in July, respectively (Stefanowicz et al. 2019). The field samples that contained Isolate 517 and Isolate 524 originated from Hel (54°36'42"N, 18°48'29"E) and Władysławowo (54°47'35"N, 18°24'69"E), respectively, and those with *Dominikia* 211 from Chałupy (54°45'31"N, 18°30'38"E) and Jastarnia (54°41'58"N, 18°40'36"E). The samples with Isolate 517 and Isolate 524 were collected by P. Niezgoda on 5 and 7 August 2021, respectively, and those with *Dominikia* 211 by J. Błaszkowski on 14 August 2012 and 11 July 2012.

The trap and single-species cultures were established and grown, spores were extracted, and mycorrhizal structures were stained as described previously (Błaszkowski et al. 2006, 2009b). Single-species cultures were established using clusters with ca. 5–30 spores connected by a common parent hypha.

### ﻿Microscopy and nomenclature

Morphological features of spore clusters and spores, as well as phenotypic and histochemical characters of spore wall layers of *Dominikia* 211, Isolate 517, and Isolate 524 were characterized based on at least 50–100 spores mounted in water, lactic acid, polyvinyl alcohol/lactic acid/glycerol (PVLG, Omar et al. 1979), and a mixture of PVLG and Melzer’s reagent (1:1, v/v). Spores for study and photography were prepared as described in Błaszkowski (2012) and Błaszkowski et al. (2012). The types of spore wall layers were defined by Błaszkowski (2012) and Walker (1983). Color names were from Kornerup and Wanscher (1983). Nomenclature of fungi and the authors of fungal names are from the Index Fungorum website http://www.indexfungorum.org/AuthorsOfFungalNames.htm. The term “glomerospores” was used for spores produced by AMF as proposed by Goto and Maia (2006).

The holotype of the new species was deposited at ZT Myc (ETH Zurich, Switzerland). Isotypes of this species and voucher specimens of the other fungi characterized here were deposited in the Laboratory of Plant Protection, Department of Environmental Management (LPPDEM), West Pomeranian University of Technology in Szczecin, Poland. In all specimens, spores were permanently mounted in PVLG and a mixture of PVLG and Melzer’s reagent (1:1, v/v) on slides.

### ﻿DNA extraction, PCR, cloning, and DNA sequencing

Genomic DNA of *Dominikia* 211, Isolate 517, and Isolate 524 was separately extracted from eight clusters of spores, each with ca. 5–30 spores formed from branches of a parent hypha. The method of processing the spores prior to PCR, conditions and primers used for PCR, as well as cloning and sequencing of PCR products to obtain 45S sequences of the isolates were as those described by Krüger et al. (2009) and Błaszkowski et al. (2021a). The sequences were deposited in GenBank (PQ459439–PQ459442, PQ459444, PQ459445, PQ464092–PQ464094).

### ﻿Phylogenetic analyses

Preliminary comparisons of 45S sequences of *Dominikia* 211, Isolate 517, and Isolate 524 showed that they are different taxa of Glomerales. Therefore, to find the position and taxonomic status of these fungi in this order, three alignments were produced using MAFFT 7 with the E-INS-i option (Katoh et al. 2019). In each alignment, the outgroup were sequences of *Entrophosporaclaroidea* as Entrophosporaceae was indicated to be a sister family to Glomerales (Błaszkowski et al. 2022b). In the 45S alignment, the ingroup contained 114 sequences of the 45S region or its part, which characterized the three main fungi analyzed here and 36 species of all genera of Glomerales, except for *Simiglomus* (see “Discussion” for the reason). The ingroup of the *rpb1* alignment consisted of 58 sequences, of which two came from *Dominikia* 211, and the others represented all species with available sequences of the *rpb1* gene. The 45S+*rpb1* alignment had all sequences of the 45S alignment concatenated with sequences of the *rpb1* alignment.

The percentage sequence divergences of *Dominikia* 211, Isolate 517, and Isolate 524 from sequences of their closest relatives were calculated in BioEdit (Hall 1999). All comparisons were performed on sequences of the same length.

The phylogenetic positions of *Dominikia* 211, Isolate 517, and Isolate 524 among the analyzed members of Glomerales were reconstructed based on Bayesian inference (BI) and maximum likelihood (ML) phylogenetic analyses of the 45S, *rpb1*, and 45S+*rpb1* alignments, performed via CIPRES Science Gateway 3.1 (Miller et al. 2010). The 45S and *rpb1* alignments were divided into five and nine partitions, respectively (45S into: 18S, ITS1, 5.8S, ITS2, 28S; *rpb1* into: five exons and four introns). In both BI and ML analyses, GTR+I+G was used as nucleotide substitution model for each nucleotide partition, as suggested by Abadi et al. (2019).

The BI reconstruction was made based on four Markov chains run over one million generations in MrBayes 3.2 (Ronquist et al. 2012), sampling every 1,000 generations, with a burn-in at 30% sampled trees. The ML phylogenetic tree inference was performed with RAxML-NG 1.0.1 (Kozlov et al. 2019), using a maximum likelihood/1000 bootstrapping run, and ML estimated proportion of invariable sites and base frequencies. The alignments and tree files were deposited as Suppl. materials. Clade and node supports were considered strong, moderate, and marginal when BI and ML support values were 0.98–0.99 and 81–99%, 0.96–0.97 and 71–80%, and 0.95 and 70%, respectively.

The phylogenetic trees were visualized and edited in FigTree ver. 1.4.4 (http://tree.bio.ed.ac.uk/software/figtree/) and MEGA6 (Tamura et al. 2013).

To detect possible other findings of *Dominikia* 211, Isolate 517 and Isolate 524, their 45S sequences were used as queries in BLASTn to retrieve environmental sequences of potentially identical species from GenBank. The sequences were selected according to the percentage of identity > 96%. Their likely identity was then verified in BI and ML analyses of the alignment with 45S+environmental sequences.

## ﻿Results

### ﻿General data and phylogeny

The alignments analyzed contained nine newly obtained sequences of the 45S region. The numbers of analyzed sequences and species/isolates, as well as the numbers of base pairs, variable, and parsimony informative sites of each of the alignments are presented in Table 1.

**Table 1.** Characteristics of the sequence alignments analyzed.

Name of alignment	No. of sequences	No. of fungal species	No. of base pairs	No. of variable sites	No. of parsimony informative sites
45S	120	39	1932	1022	937
*rpb1*	59	31	2841	1350	1002
45S+*rpb1*	120	39	4441	2244	1855

**Figure 1.**
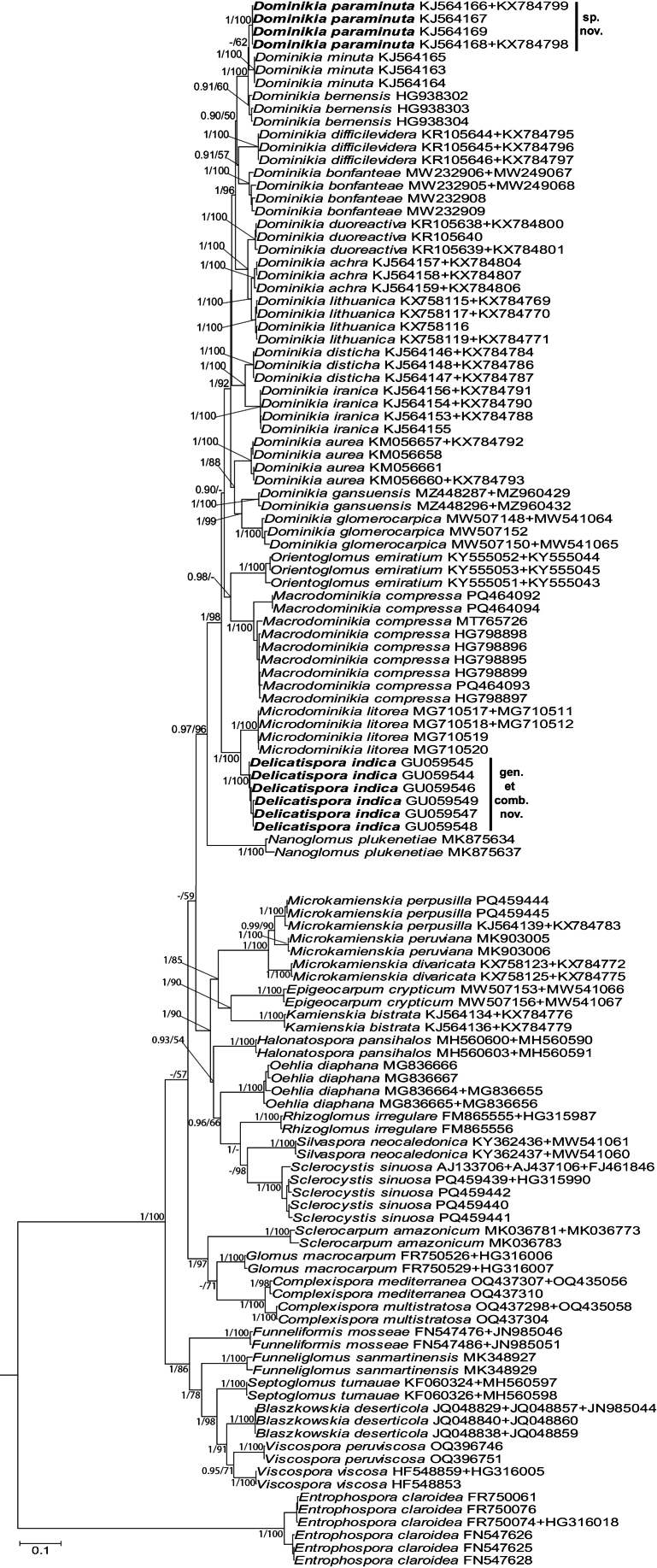
50% majority-rule consensus tree from the Bayesian analysis of sequences of 45S nuc rDNA concatenated with *rpb1* sequences of *Macrodominikiacompressa* (= Isolate 517), *Delicatisporaindica*, *Dominikiaparaminuta*, 33 other species of Glomerales, as well as *Entrophosporaclaroidea* serving as outgroup. The new genera and species are in bold font. The Bayesian posterior probabilities ≥ 0.90 and ML bootstrap values ≥ 50% are shown near the branches, respectively. Bar indicates 0.1 expected change per site per branch.

The topologies of the trees with 45S and 45S+*rpb1* sequences generated following the BI and ML analyses were identical (Fig. 1, Suppl. material 1). Small and insignificant differences in the topology of the *rpb1* tree compared to the topologies of the 45S and 45S+*rpb1* trees resulted from the lack of *rpb1* sequences of eight of the 39 analyzed species (Suppl. material 2).

*Dominikiaindica* was placed in an autonomous generic clade, the sister of which was a clade populated by *Microdominikialitorea* (Fig. 1, Suppl. material 1). Both clades and the node connecting them were fully or strongly supported in BI and ML analyses.

*Dominikia* 211 occupied a new, fully supported species clade, sister to the *D.minuta* clade, which obtained full BI and ML supports (Fig. 1, Suppl. material 1). None of the BI and ML analyses supported the node linking the two clades. In the *rpb1* tree, *Dominikia* 211 grouped in a clade neighboring with a clade with *D.duoreactiva*, *D.lithuanica*, and *D.achra* (Suppl. material 2). Both clades obtained very high supports (BI = 1.0, ML = 98%).

Isolate 517 and *Macrodominikiacompressa* formed an autonomous clade at the rank of genus in a sister position to a clade with *Orientoglomusemiratium*. Both clades obtained full BI (= 1.0) and ML (= 100%) supports (Fig. 1, Suppl. material 1). Instead, the node connecting the two clades was supported only by the BI analysis of the 45S+*rpb1* sequences (Fig. 1).

Isolate 524 clustered with *Microkamienskiaperpusilla* in a clade with full BI and ML supports (Fig. 1, Suppl. material 1).

The genetic distance between the 45S sequences of *D.indica* and *Mi.litorea* ranged from 18.0 to 19.1%. The range of the sequence divergences between *Dominikia* 211 and *D.minuta* was 2.4–2.7%. The sequences of Isolate 517 and *M.compressa* versus *D.emiratia* differed by 16.7–17.3%.

### ﻿Taxonomy

The phylogenetic analyses and sequence comparisons described above clearly indicated that (i) *D.indica* with the GU059544–GU059549 sequences should belong to a new genus in Dominikiaceae, (ii) Isolate 211 is a new *Dominikia* species, (iii) Isolate 517 and the species originally described as *G.compressum* (Oehl et al. 2014), later transferred first to *Dominikia* (Oehl et al. 2015), and recently to *Macrodominikia* under the name *M.compressa* in Dominikiaceae (Silva et al. 2024), are conspecific, and (iv) Isolate 524 is conspecific with *Microkamienskiaperpusilla* in Kamienskiaceae (Silva et al. 2024). Consequently, (i) *D.indica* was transferred to *Delicatispora* gen. nov. and renamed *De.indica* comb. nov. in Dominikiaceae, (ii) Isolate 211 was described as *Dominikiaparaminuta* sp. nov., (iii) the morphological description of *M.compressa* was emended based on new data obtained from analyses of Isolate 517, and (iv) the morphology of the *Mk.perpusilla* specimens considered here was compared with that presented in the original description of this species (Błaszkowski et al. 2009a), as well as the occurrence of *Mk.perpusilla* in the world was discussed.

### ﻿Descriptions of a new genus and combination, a new species, an emendation of *Macrodominikiacompressa*, and notes on *Microkamienskiaperpusilla*

#### ﻿ Delicatispora

Taxon classificationFungiGlomeralesDominikiaceae

Błaszk., Niezgoda & B.T.Goto gen. nov.

DB578615-C570-5B27-BA46-D29A15810478

856187

Fig. 1 , Suppl. material 1

##### Etymology.

Latin, *Delicati* and *spora*, referring to the delicate spores produced by the type species of the genus.

##### Type genus.

*Delicatisporaindica* (Błaszk., Wubet & Harikumar), Błaszk., Niezgoda & B.T.Goto, comb. nov.

##### Basionym.

*Glomusindicum* Błaszk., Wubet & Harikumar.

##### Synonym.

*Dominikiaindica* (Błaszk., Wubet & Harikumar) Błaszk., Chwat & Kovács.

##### Diagnosis.

Differs from other genera of Glomerales in nucleotide composition of sequences of the 45S nuc rDNA region (see “Discussion” for details).

##### Genus description.

As that in Błaszkowski et al. (2010b).

#### ﻿ Delicatispora indica

Taxon classificationFungiGlomeralesDominikiaceae

(Błaszk., Wubet & Harikumar) Błaszk., Niezgoda & B.T.Goto comb. nov.

60062287-D34E-5DBF-8C6F-D9F6B3F88A50

856189

Fig. 1 , Suppl. material 1

##### Etymology.

Latin, referring to India where this species was originally found.

##### Specimens examined.

Poland. Spores from single-species cultures established from spores extracted from trap pot cultures inoculated with rhizosphere soil and root fragments of *Euphorbiaheterophylla* L. from coastal sands of Alappuzha in Kerala State of South India (90°55'N, 76.0°46'E) and *Lactucasativa* L. cultivated in Asmara, Eritrea, North East Africa (15°28'N, 38°55'E), 10 Apr 2009, J. Błaszkowski (holotype: slide with spores no. LPPDSE 3113; isotypes: slides with spores nos. LPPDSE 3108–3112 and 3114–3133), and two slides at OSC.

##### Diagnosis.

As that of *Delicatispora* (see above).

##### Description.

As that in Błaszkowski et al. (2010b).

##### Ecology and distribution.

Originally found in two trap pot cultures inoculated with rhizosphere soils and root fragments of *E.heterophylla* from coastal sands of Alappuzha in Kerala State of South India and *L.sativa* cultivated in Asmara, Eritrea, Northeast Africa. The geographic positions of the sampled sites, physicochemical properties of their soils, and features of mycorrhizal structures formed in single-species cultures of this fungus, are given in Błaszkowski et al. (2010b). Based on ≥ 98% SSU rDNA sequence identity to uncultured AMF sequences available in public databases, suggested to have also been present in various states of the USA, Estonia, and Australia (Błaszkowski et al. 2010b). In the USA, also found associated with roots of *Panicumvirgatum* L. in Wisconsin, as resulted from phylogenetic analyses of our 45S alignment with the MT765488, MT765651, and MT765457 environmental sequences with > 96% identity to 45S sequences of *D.indica* (data not shown).

#### ﻿ Dominikia paraminuta

Taxon classificationFungiGlomeralesDominikiaceae

Błaszk., Niezgoda & B.T.Goto sp. nov.

DE6C09F5-C5D6-58FC-B190-6F6B7F7D5A93

856191

Fig. 2A–H

##### Specimens examined.

Poland. Pomeranian Province, spores from single-species cultures established from spores extracted from trap pot cultures inoculated with rhizosphere soil and root fragments of *Ammophilaarenaria* from the Hel Peninsula maritime dunes (54°45'31"N, 18°30'38"E and 54°41'58"N, 18°40'36"E), 14 Aug 2012, J. Błaszkowski (***holotype***: slide with spores Z+ZT Myc 0067480; ***isotypes***: slides with spores nos. 3979–3987, LPPDSE).

##### Etymology.

Latin, *paraminuta*, referring to *D. minuta*, the phylogenetic sister of this new species.

##### Diagnosis.

Differs from *D.minuta*, the phylogenetic sister, in (i) morphometric features of spores and their spore wall, (ii) the spore wall structure, (iii) phenotypic properties of spore wall layer 1, and (iv) nucleotide composition of sequences of the 45S nuc rDNA region (see “Discussion” for details).

##### Description.

**Figure 2.**
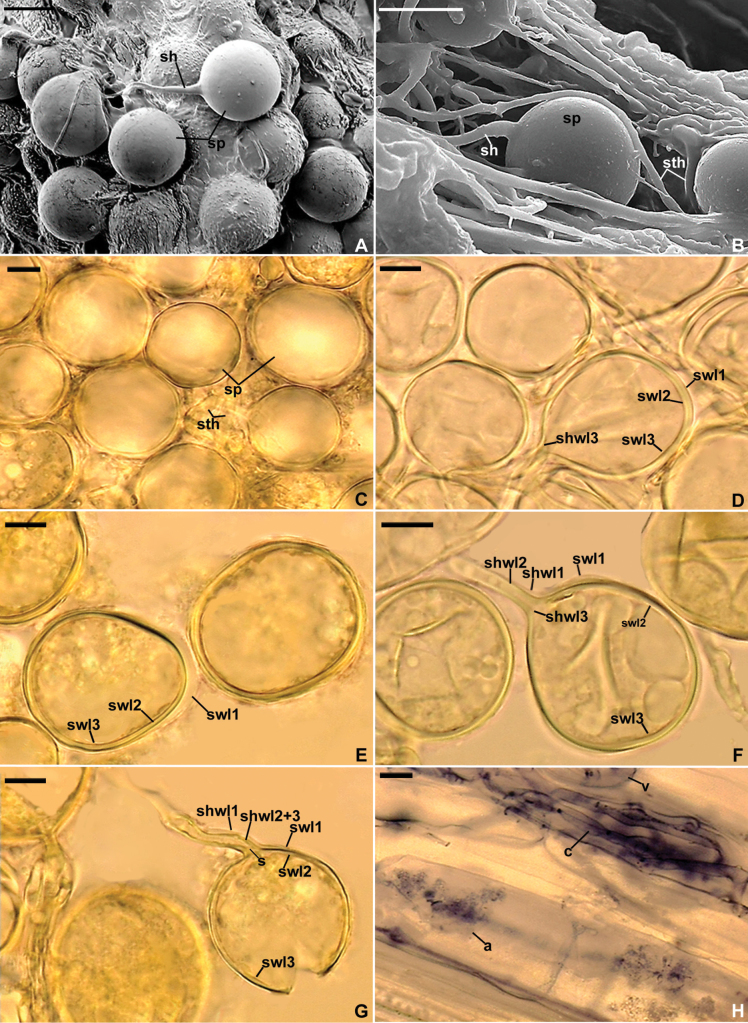
*Dominikiaparaminuta***A–C** cluster of spores (sp) with subtending hypha (sh), and sterile hyphae (sth) **D–G** spore wall layers (swl) 1–3 continuous with subtending hyphal wall layers (shwl) 1–3; septum (s) connecting the inner surfaces of shwl2 is indicated in **G, H** arbuscule (a), vesicle (v), and coiled hyphae (c) in mycorrhizal root of *Plantagolanceolata* stained in 0.1% Trypan blue **C, D, H** spores and mycorrhizal structures in PVLG**E, F, G** spores in PVLG+Melzer’s reagent **A, B** scanning electron microscopy **C–H** differential interference microscopy. Scale bars: 20 μm (**A**); 20 μm (**B**); 10 μm (**C–H**).

Forming loose to compact hypogeous clusters with five to ca. 25 randomly distributed spores and sterile hyphae (Fig. 2A–D). Spores glomoid, arising blastically at tips of subtending hyphae (Fig. 2A, B, F, G) branched from a parent hypha continuous with a mycorrhizal extraradical hypha. ***Spores*** hyaline; globose to subglobose; (23–)31(–39) µm diam; rarely ovoid to oblong, 14–33 × 26–71 µm; with one subtending hypha (Fig. 2A–G). ***Spore wall*** composed of three hyaline layers (layers 1–3; Fig. 2D–G). Layer 1, forming the spore surface, uniform (without visible sublayers), semi-permanent, (0.8–)1.3(–2.0) µm thick when smooth, often with local thickenings, (1.2–)2.1(–3.8) µm thick, randomly distributed on the spore surface, rarely strongly or completely sloughed off in aged spores (Fig. 2D–G). Layer 2 laminate, permanent, smooth, (1.0–)1.4(–2.0) µm thick, consisting of very thin, < 0.5 µm, sublayers tightly adherent to and not separating from each other even in vigorously crushed spores (Fig. 2D–G). Layer 3 uniform, permanent, smooth, ca. 0.6–0.8 µm thick, usually tightly adherent to the inner surface of layer 2 and, therefore, difficult to detect. Layers 1–3 do not stain in Melzer’s reagent (Fig. 2E–G). ***Subtending hypha*** hyaline; straight or recurved, usually cylindrical to funnel-shaped, rarely slightly constricted at the spore base, (3.8–)5.7(–9.5) µm wide at the spore base (Fig. 2A, B, F, G). ***Wall of subtending hypha*** hyaline; (1.6–)2.2(–2.9) µm thick at the spore base; composed of three layers continuous with spore wall layers 1–3 (Fig. 2F, G). ***Pore*** (1.0–)1.9(–5.8) µm diam, usually open (Fig. 2F), very rarely occluded by a curved septum connecting the inner surfaces of subtending hyphal wall layer 3; septum 0.4–0.6 µm thick, located ca. 2.0 µm below the spore base (Fig. 2G). ***Sterile hyphae*** hyaline, (2.8–)4.4(–5.2) µm wide (Fig. 2B, C). ***Germination*** unknown.

##### Ecology and distribution.

In the field, *D.paraminuta* probably lived in arbuscular mycorrhizal symbiosis with roots of *A.arenaria* that colonized maritime sand dunes near Chałupy and Jastarnia on the Hel Peninsula, northern Poland. However, no molecular analyses were performed to confirm this assumption. In single-species cultures with *P.lanceolata* as host plant, *D.paraminuta* formed mycorrhiza with arbuscules, vesicles, and intra- and extraradical hyphae (Fig. 2H). These structures stained clearly [pale violet (16A3) to deep violet (16E8)] in 0.1% Trypan blue. Phylogenetic analyses with the 45S alignment used in this study and environmental sequences with > 96% identity to 45S sequences of *D.paraminuta*, revealed by BLASTn, indicated that *D.paraminuta* was previously recognized in grasslands and unspecified sites in China (data not shown).

#### ﻿ Macrodominikia compressa

Taxon classificationFungiGlomeralesDominikiaceae

(Sieverd., Oehl, Palenz., Sánchez-Castro & G.A.Silva) emend. Błaszk., Niezgoda & B.T.Goto

65C2AB47-7765-5040-9DA8-BCE5E0056791

Figs 3A–H , 4A, B

##### Specimens examined.

Poland. Pomeranian Province, spores from a trap pot culture inoculated with rhizosphere soil and root fragments of *Ammophilaarenaria* from the Hel Peninsula maritime dunes (54°36'42"N, 18°48'29"E), 5 Aug 2021, P. Niezgoda (slides with spores nos. 3988–3990, LPPDSE). Switzerland. Two slides with holotype sporocarps and spores deposited under the accession number Z+ZT Myc 52538. Brazil. Thirty-three isotypic spores deposited under the URM85721 accession number.

##### Diagnosis.

Differs from other genera of Dominikiaceae in (i) having subtending hyphae with a strong bend and locally very narrow lumen due to large thickening present on the inner surfaces of the subtending hyphal walls and (ii) nucleotide composition of sequences of the 45S nuc rDNA region (see “Discussion” for details).

##### Description.

**Figure 3.**
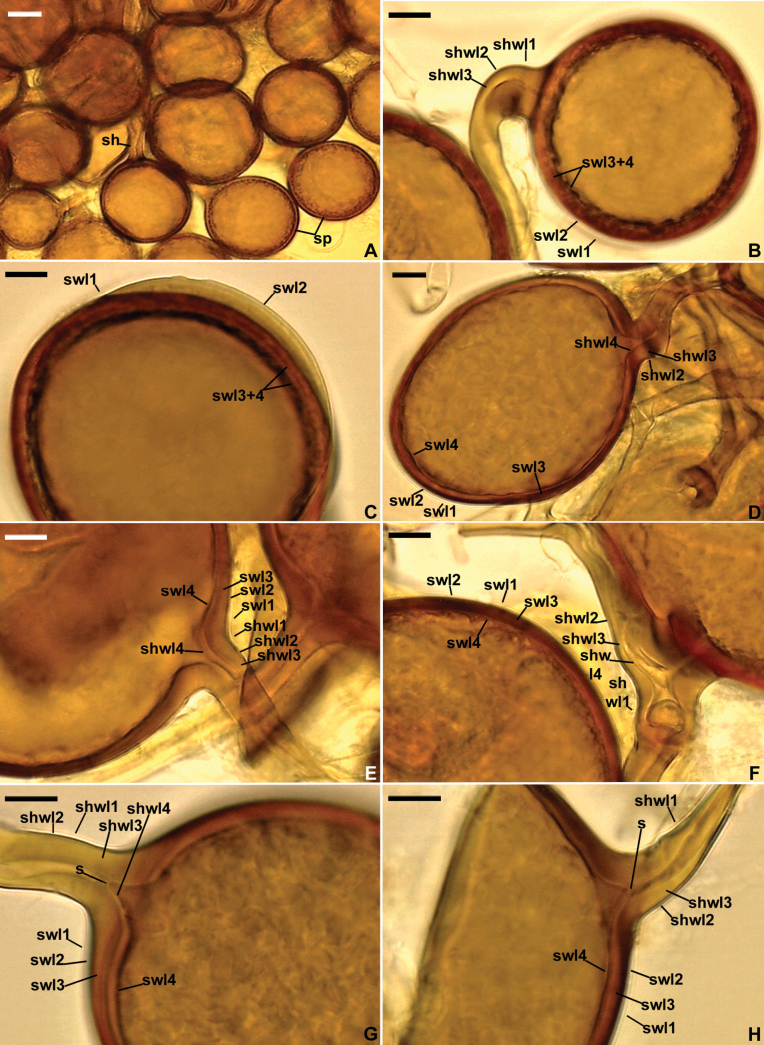
*Macrodominikiacompressa***A** cluster of spores (sp) with subtending hypha (sh) **B** spore wall layers (swl) 1–4 and subtending hyphal wall layers (shwl) 1–3; shwl4 is not visible **C** spore wall layers (swl) 1–4 **D–H** spore wall layers (swl) 1–4 continuous with subtending hyphal wall layers (shwl) 1–4; septum (s) formed by shwl4 is indicated **A** spores in PVLG**B–H** spores in PVLG+Melzer’s reagent **A–H** differential interference microscopy. Scale bars: 20 μm (**A**); 10 μm (**B–H**).

**Figure 4.**
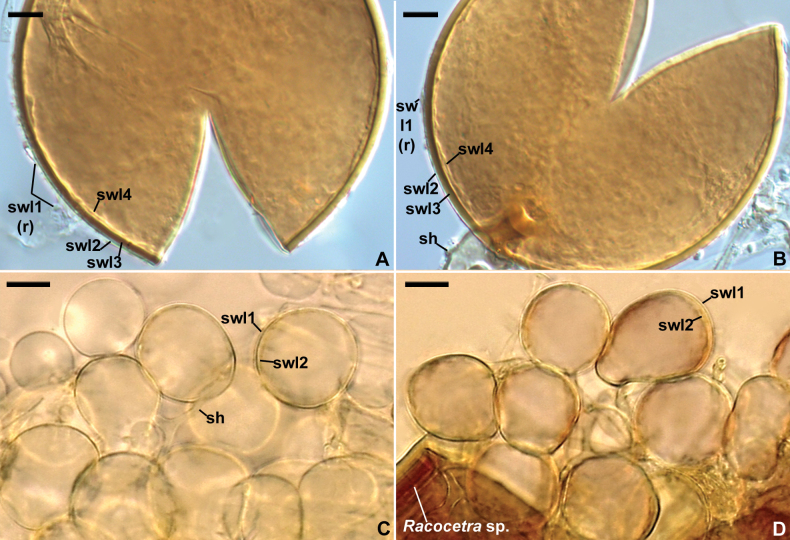
*Macrodominikiacompressa***A** remnants (r) of spore wall layer (swl) 1 and spore wall layers (swl) 2–4; spore subtending hypha (sh) is indicated in **B***Microkamienskiaperpusilla***C** cluster of spores formed in the growth substrate of a single-species culture; swl1 and 2 and spore subtending hypha (sh) are indicated **D** cluster of spores produced inside a spore of *Racocetra* species extracted from a trap culture; swl1 and 2 are indicated **A, B, C** spores in PVLG**D** spores in PVLG+Melzer’s reagent **A–D** differential interference microscopy. Scale bars: 10 μm (**A–D**).

Forming loose to compact hypogeous clusters with 3–33 randomly distributed spores (Fig. 3A), and spores singly in the soil. Spores glomoid, arising blastically at tips of subtending hyphae (Figs 3B, D–H, 4B) either branched from a parent hypha continuous with a mycorrhizal extraradical hypha (spores in clusters), or directly continuous with a mycorrhizal extraradical hypha (single spores). ***Spores*** pale yellow (4A3) to brownish yellow (5C8); globose to subglobose; (19–)62(–100) µm diam; rarely ovoid to oblong 22–81 × 66–134, to irregular; with one subtending hypha (Figs 3A–H, 4A, B). ***Spore wall*** composed of four layers (layers 1–4; Figs 3B–H, 4A, B). Layer 1, forming the spore surface, evanescent, flexible, hyaline, (0.6–)0.9(–1.4) µm thick, usually slightly swelling in PVLG and then easier to detect (Fig. 3B–H), occasionally strongly or completely sloughed off in aged spores (Fig. 4A, B). Layer 2 uniform (without visible sublayers), permanent, flexible to semi-flexible, smooth, hyaline, (0.8–)1.2(–1.5) µm thick, tightly adherent to layer 3 (Figs 3B–H, 4A, B). Layer 3 laminate, semi-rigid, smooth, pale yellow (4A3) to brownish yellow (5C8), (2.8–)3.8(–6.3) µm thick, consisting of very thin, < 0.5 µm thick, sublayers tightly adherent to and not separating from each other even in vigorously crushed spores (Figs 3B–H, 4A, B). Layer 4 uniform, flexible to semi-flexible, smooth, concolorous with or slightly lighter than layer 3, (0.8–)1.0(–1.3) µm thick, usually only slightly separating from the lower surface of layer 3 even in vigorously crushed spores (Figs 3B–H, 4A, B). Layers 1–4 do not stain in Melzer’s reagent (Fig. 3B–H). ***Subtending hypha*** concolorous with or slightly lighter than the spores; straight or recurved, usually cylindrical to funnel-shaped, rarely slightly constricted at the spore base, (9.6–)14.2(–20.4) µm wide at the spore base (Figs 3A, B, D–H, 4B). ***Walls of subtending hypha*** concolorous with or slightly lighter than the spores; usually with thickenings of unequal size, (5.8–)7.2(–11.0) µm vs. (4.0–)5.1(–6.0) µm thick, present on the inner, opposite surfaces of the walls, making the subtending hyphal lumen more or less curved and narrow when seen in a plan view (Fig. 3A, D, E, H); less often, both walls have the same or similar thickness, (4.0–)6.3(–8.1) µm (Fig. 3G); subtending hyphal walls composed of four layers continuous with spore wall layers 1–4 (Fig. 3B, D, E–H). ***Pore*** (1.0–)2.4(–7.0) µm diam, open (Fig. 3D, E) or occluded by a curved septum continuous with spore wall layer 4; septum 0.6–1.0 µm thick, located at or up to 8.2 µm below the spore base (Fig. 3G, H); subtending hyphal lumen rarely occluded by an additional septum located up to 22 µm below the spore base. ***Sterile hyphae*** hyaline, (1.8–)4.2(–5.6) µm wide. ***Germination*** unknown.

##### Ecology and distribution.

Results from our studies conclude that in the field *M.compressa* probably lived in arbuscular mycorrhizal symbiosis with roots of *A.arenaria* that colonized maritime dunes near Hel on the Hel Peninsula in northern Poland. However, no molecular analyses were performed to confirm this assumption. In a trap pot culture, *M.compressa* produced abundant spore communities. Oehl et al. (2014) found this species, as *G.compressum*, in ten locations in Switzerland, southwestern Germany, and northeastern France. It occurred in grasslands and crop rotation systems located at altitudes between 230–1505 m asl., with soils with a wide range of pH (5.7–8.0), organic carbon (9.8–45.8 g kg^-1^), and different levels of plant available phosphate. There is no sequence in GenBank with > 96% identity to the 45S sequence *M.compressa*, which would suggest the presence of this species in other regions of the world.

### ﻿Notes on *Microkamienskiaperpusilla*

#### ﻿ Microkamienskia perpusilla

Taxon classificationFungiGlomeralesKamienskiaceae

(Błaszk. & Kovács) Corazon-Guivin, G.A.Silva & Oehl

93569CDE-C683-5068-9D8C-34906ADFADF8

Fig. 4C, D

##### Basionym.

*Glomusperpusillum* Błaszk. & Kovács.

##### Synonym.

*Kamienskiaperpusilla* (Błaszk. & Kovács) Błaszk., Chwat & Kovács.

##### Specimens examined.

Poland. Pomeranian Province, spores from single-species cultures established from spores extracted from a trap pot culture inoculated with rhizosphere soil and root fragments of *Ammophilaarenaria* from the Hel Peninsula maritime dunes (54°47'35"N, 18°24'69"E), 7 Aug 2021, P. Niezgoda (slides with spores nos. 3991–3996, LPPDSE).

##### Diagnosis.

Differs from *Mk.peruviana*, the phylogenetic sister (Fig. 1), in (i) morphometric features of the spore wall, the spore subtending hypha, and the pore connecting the subtending hyphal lumen with the spore interior, (ii) phenotypic properties of spore wall layer 1 and subtending hyphal wall layer 1, and (iii) nucleotide composition of sequences of the 45S nuc rDNA region (Błaszkowski et al. 2009a; Corazon-Guivin et al. 2019b; see “Discussion” for details).

##### Notes.

The morphological features of Isolate 524 (Fig. 4C, D), here recognized to be conspecific with *Mk.perpusilla*, were nearly identical to those originally defined for *G.perpusillum* (Błaszkowski et al. 2009a). The only discrepancy was the lack of plasticity and contractibility of spore wall layer 2 in Isolate 524, which were considered unique features of *G.perpusillum*.

## ﻿Discussion

The results of the morphological and phylogenetic analyses discussed above significantly improved the knowledge of two AMF species originally described as *Glomuscompressum* and *G.indicum* (Błaszkowski et al. 2010b; Oehl et al. 2014). Apart from supporting the validity of the creation of a new genus, *Macrodominikia*, with *M.compressa* comb. nov. in the new family Dominikiaceae (Silva et al. 2024), these analyses also allowed us to (i) emend the morphological description of this species and (ii) transfer *G.indicum* to a new genus, *Delicatispora*, with *De.indica* comb. nov. Moreover, these analyses (i) confirmed our suspicions about the novelty of the fungus informally named Isolate 211 and (ii) proved that it is identical to the fungus provided with an incorrect and, consequently, misleading name, *Dominikiaindica* strain 211, which erroneously suggested its conspecificity with *D.indica*. Finally, based on morphological and phylogenetic analyses, we found a new site of occurrence of *Mk.perpusilla* and minor phenotypic and histochemical differences in spore wall layer 2 of the newly collected specimens compared to the phenotypic and histochemical characters of spore wall layer 2 characterized in the original description of this species.

In addition to the indications of phylogenetic analyses (Fig. 1, Suppl. material 1), the distinctiveness of *De.indica* was strongly confirmed by comparisons of its sequences with the sequences of representatives of all other genera of Glomerales. The magnitude of the sequence divergences of *De.indica* from those of its sister genus (*Microdominikia*), which was 26.2–26.5%, was equivalent to or significantly greater than sequence divergences between most other genera of Glomerales shown in Fig. 1. Only the sequence divergences between the sister or closest generic clades *Complexispora* vs. *Funneliformis*, *Dominikia* vs. *Orientoglomus*, *Glomus* vs. *Sclerocarpum*, *Oehlia* vs. *Rhizoglomus* were higher and amounted to 39.0%, 32.2%, 35.0%, and 34.9%, respectively.

Morphologically, *Delicatisporaindica* is almost identical to *Dominikiabonfanteae*. Both species produce spores in loose clusters, these spores are hyaline, overlap in size, when globose, and their spore wall consists of two layers with almost identical phenotypic and histochemical properties (Błaszkowski et al. 2010b, 2021c). However, the 45S sequences of these two species differ by 13.3–14.6%. Thus, the lack of a synapomorphic feature in *De.indica* and the vast majority of other species producing glomoid spores, i.e., ca. 65% of all representatives of Glomeromycota, as well as the recently observed crypticity in some Glomeromycota species (Błaszkowski et al. 2021a, 2022b) indicate that identifying and classifying glomoid spore-producing members of Glomeromycota solely on the basis of morphology is difficult, uncertain, or impossible (Goto et al. 2024).

As mentioned above, our analyzes demonstrated that Isolate 211 is a new species, here described as *Dominikiaparaminuta*, and its phylogenetic sister is *D.minuta* (Fig. 1, Suppl. materials 1, 2). Both species differ clearly in terms of the morphometric features of spores and their spore wall, the spore wall structure, and the phenotypic properties of spore wall layer 1, forming the spore surface. *Dominikiaminuta* spores are 1.3–1.7-fold larger when globose and their spore wall is 2.1–2.6-fold thinner (Błaszkowski et al. 2000). The most significant differences between these species lie in their spore wall structure and spore wall layer 1. In *D.minuta*, the spore wall consists of two layers and spore wall layer 1 is permanent, always present regardless of the age of the spores, and smooth. In *D.paraminuta*, the spore wall has three layers and spore wall layer 1 is semi-permanent and sometimes strongly or completely sloughed off (Fig. 2E). Moreover, its upper surface is often ornamented with clearly visible thickenings (Fig. 2D–F). Importantly, in young *D.minuta* spores, spore wall layer 1 is flexible and locally swells in PVLG, which causes its strong separation from the upper surface of the laminate layer 2 (Błaszkowski et al. 2000; Błaszkowski 2012; https://zor.zut.edu.pl/Glomeromycota_2/Glomus%20minutum.html). In older spores, this layer becomes rigid, fragile, and often falls apart in crushed spores. None of these features are present in spore wall layer 1 of *D.paraminuta*.

Oehl et al. (2014) characterized *M.compressa*, under the name *Glomuscompressum*, as producing spores with a two-layered spore wall: an evanescent, hyaline outer layer, which can slightly expand in PVLG, and a laminate, colored inner layer. Properties of the outer layer sensu Oehl et al. (2014) exactly match the properties of spore wall layer 1 of our specimens of *M.compressa* (Figs 3B–H, 4A, B). However, spore wall layer 1 of our specimens surrounds a uniform, permanent, hyaline spore wall layer 2 (Figs 3B–H, 4A, B). This layer 2 is visible in fig. 4 by Oehl et al. (2014) and was probably considered the non-swellable part of the outer evanescent layer. Also, spore wall layer 4 of *M.compressa*, which we found in our specimens (Figs 3B–H, 4A, B), is clearly visible in figs 4–6 by Oehl et al. (2014). It was probably treated as the innermost lamina of the laminate inner layer, which often separates from the other laminae of this type of layer, especially in vigorously crushed spores. In our specimens, this layer is uniform (without visible laminae) and thicker than, e.g., the lamina of the laminate layer 3 visible in Fig. 3D, E. These four spore wall layers are also well visible on holotype and isotypes deposited by the authors of this species under the accession number Z+ZT Myc 52538, and URM85721, respectively (pers. observ., Fig. 4A, B).

We agree with Oehl et al. (2014) and Silva et al. (2024) that *M.compressa* has a distinct morphological feature that could be considered synapomorphy, defining a unique monophyletic group. It is a strongly bent and locally very narrow lumen of the spore subtending hypha when seen in a plan view due to large thickenings present on the inner surfaces of its walls (Fig. 3B, D, F).

Our phylogenetic analyses and comparisons of 45S sequences of Isolate 524 with those available in GenBank indicated that this isolate represents *Microkamienskiaperpusilla*, originally discovered in maritime dunes of Italy (Błaszkowski et al. 2009a), and that the maritime dunes located near Hel, northern Poland, are the second site in the world, in which this species has been found so far. The lack of plasticity and contractibility of spore wall layer 2 in Polish *Mk.perpusilla* specimens (Fig. 4C, D), in contrast to Italian specimens where these features were considered unique to members of Glomerales (Błaszkowski et al. 2009a), suggests that the phylogenetic informativeness of these characters is low or non-existent.

Worth mentioning, our phylogenetic analyses for the first time included continuous 45S sequences of *Sclerocystissinuosa* of a length of ca. 1570 bp (Fig. 1, Suppl. material 1), which we obtained from sporocarps found in Benin, Africa. This will certainly significantly improve the reliability of reconstructing the phylogenetic positions of Glomerales members because the available so far rDNA sequences of *S.sinuosa* originated only from part of the 45S segment, i.e., the 18S gene (partial), the 18S-ITS1-5.8S-ITS2 region, or the 28S gene (partial). The species resolution of glomeromycotan 18S, ITS1, and ITS2 sequences usually is low and often does not separate closely related species (Krüger et al. 2009; pers. observ.). In addition, short sequences, such as the previously available *S.sinuosa* 28S sequences of a length of 691–714 bp (FJ461846, MT832185, MT832186), may cause ambiguities because the phylogenetic signal they contain is small and leads to little node supports in phylogenetic trees (Redecker et al. 2013, pers. observ.).

It is widely accepted that the reliability of fungal phylogenies reconstructed based on sequences derived from multiple loci, including at least one protein-coding locus, is significantly greater than the reliability of phylogenies obtained from single-locus sequence analyses (Chethana et al. 2021). Unfortunately, our numerous attempts to obtain *rpb1* sequences for Isolate 517 (= *M.compressa*) and *Delicatisporaindica* failed. This is in strong contrast to the very high success rate of obtaining *rpb1* sequences in our laboratory to date, as evidenced by the fact that ca. 68% of all the glomeromycotan protein-coding *rpb1* gene sequences deposited in GenBank were obtained by J. Błaszkowski and his co-workers. Despite this failure, we believe that the *M.compressa* and *De.indica* phylogenies are reliable because we found no conflict between the phylogenies we have reconstructed over the past eight years from analyses of 45S, *rpb1*, and 45S+*rpb1* sequences of numerous AMF species with different evolutionary relationships (Al-Yahya’ei et. al. 2016; Błaszkowski et al. 2016, 2018a, 2018b, 2018c, 2021a, 2021b, 2021c, 2022a, 2022b, 2023, 2024; Symanczik et al. 2018, Yu et al. 2022; Niezgoda et al. 2024).

The reason for not including the monospecific genus *Simiglomus* with *S.hoi* in our phylogenetic analyses was that this species is molecularly characterized so far only by two sequences of the 18S gene (Oehl et al. 2011). In our alignments, this gene was represented by only ca. 240 base pairs, the phylogenetic informativeness of which certainly is too low to show a reliable taxonomic position of *S.hoi*.

## Supplementary Material

XML Treatment for Delicatispora

XML Treatment for Delicatispora indica

XML Treatment for Dominikia paraminuta

XML Treatment for Macrodominikia compressa

XML Treatment for Microkamienskia perpusilla

## ﻿Additional information

### Author ORCIDs

Janusz Błaszkowski https://orcid.org/0000-0003-3688-164X

Szymon Zubek https://orcid.org/0000-0003-1338-9572

Paweł Milczarski https://orcid.org/0000-0003-2622-8938

Ryszard Malinowski https://orcid.org/0000-0003-3413-2653

Piotr Niezgoda https://orcid.org/0000-0002-0560-6154

Bruno Tomio Goto https://orcid.org/0000-0001-6157-4954

### Data availability

Datasets generated during and/or analyzed during the current study are available from the corresponding author upon request.

## Supplementary materials

Supplementary material 1 50% majority-rule consensus tree from the Bayesian analysis of sequences of 45S nuc rDNA sequences of *Macrodominikiacompressa*, *Delicatisporaindica*, *Dominikiaparaminuta*, 33 other species of Glomerales, as well as *Entrophosporaclaroidea* serving as outgroup Data type zip Explanation note The new genera and species are in bold font. The Bayesian posterior probabilities ≥ 0.90 and ML bootstrap values ≥ 50% are shown near the branches, respectively. Bar indicates 0.1 expected change per site per branch. https://binary.pensoft.net/file/1229339This dataset is made available under the Open Database License (http://opendatacommons.org/licenses/odbl/1.0/). The Open Database License (ODbL) is a license agreement intended to allow users to freely share, modify, and use this Dataset while maintaining this same freedom for others, provided that the original source and author(s) are credited.Janusz Błaszkowski, Szymon Zubek, Paweł Milczarski, Ryszard Malinowski, Piotr Niezgoda, Bruno Tomio Goto

Supplementary material 2 50% majority-rule consensus tree from the Bayesian analysis of sequences of *rpb1* sequences of *Dominikiaparaminuta*, 29 other species of Glomerales, as well as *Entrophosporaclaroidea* serving as outgroup Data type pdf Explanation note The new genera and species are in bold font. The Bayesian posterior probabilities ≥ 0.90 and ML bootstrap values ≥ 50% are shown near the branches, respectively. Bar indicates 0.1 expected change per site per branch. https://binary.pensoft.net/file/1229340This dataset is made available under the Open Database License (http://opendatacommons.org/licenses/odbl/1.0/). The Open Database License (ODbL) is a license agreement intended to allow users to freely share, modify, and use this Dataset while maintaining this same freedom for others, provided that the original source and author(s) are credited.Janusz Błaszkowski, Szymon Zubek, Paweł Milczarski, Ryszard Malinowski, Piotr Niezgoda, Bruno Tomio Goto

## References

[B1] Abadi S Azouri D Pupko T Mayrose I (2019) Model selection may not be a mandatory step for phylogeny reconstruction. Nature Communications 10(1): 934. 10.1038/s41467-019-08822-w PMC6389923 30804347

[B2] Al-Yahya’ei MN Mullath SK AlDhaheri LA Kozłowska A Błaszkowski J (2017) *Dominikiaemiratia* and *Rhizoglomusdunense*, two new species in the Glomeromycota. Botany 95(7): 629–639. 10.1139/cjb-2016-0294

[B3] Błaszkowski J (2012) Glomeromycota. W. Szafer Institute of Botany, Polish Academy of Sciences, Kraków, 303 pp.

[B4] Błaszkowski J Tadych M Madej M (2000) *Glomusminutum*, a new species in Glomales (Zygomycetes) from Poland. Mycotaxon 76: 187–195.

[B5] Błaszkowski J Renker C Buscot F (2006) *Glomusdrummondii* and *G.walkeri*, two new species of arbuscular mycorrhizal fungi (Glomeromycota). Mycological Research 110(5): 555–566. 10.1016/j.mycres.2006.02.006 16769509

[B6] Błaszkowski J Kovács GM Balázs T (2009a) *Glomusperpusillum*, a new arbuscular mycorrhizal fungus. Mycologia 101(2): 245–253. 10.3852/08-087 19397199

[B7] Błaszkowski J Ryszka P Oehl F Koegel S Wiemken A Kovács GM Redecker D (2009b) *Glomusachrum and G. bistratum*, two new species of arbuscular mycorrhizal fungi (Glomeromycota) found in maritime sand dunes. Botany 87(3): 260–271. 10.1139/B08-138

[B8] Błaszkowski J Kovács GM Balázs T Orłowska E Sadravi M Wubet T Buscot F (2010a) *Glomusafricanum* and *G.iranicum*, two new species of arbuscular mycorrhizal fungi (Glomeromycota). Mycologia 102(6): 1450–1462. 10.3852/09-302 20943558

[B9] Błaszkowski J Wubet T Harikumar VS Ryszka P Buscot F (2010b) *Glomusindicum*, a new arbuscular mycorrhizal fungus. Botany 88(2): 132–143. 10.1139/B09-104

[B10] Błaszkowski J Kovács GM Gáspár BK Balázs TK Buscot F Ryszka P (2012) The arbuscular mycorrhizal *Paraglomusmajewskii* sp. nov. represents a new distinct basal lineage in Paraglomeraceae (Glomeromycota). Mycologia 104: 148–156. 10.3852/10-430 21914831

[B11] Błaszkowski J Chwat G Góralska A Ryszka P Kovács GM (2015a) Two new genera, *Dominikia* and *Kamienskia*, and *D.disticha* sp. nov. in Glomeromycota. Nova Hedwigia 100(1–2): 225–238. 10.1127/nova_hedwigia/2014/0216

[B12] Błaszkowski J Chwat G Symanczik S Góralska A (2015b) *Dominikiaduoreactiva* and *D.difficilevidera*, two new species in the Glomeromycota. Botany 93: 389–396. 10.1139/cjb-2015-0016

[B13] Błaszkowski J Chwat G Góralska A (2016) *Dominikialithuanica* and *Kamienskiadivaricata*: New species in the Glomeromycota. Botany 94(12): 1075–1085. 10.1139/cjb-2016-0167

[B14] Błaszkowski J Kozłowska A Niezgoda P Goto BT Dalpé Y (2018a) A new genus, *Oehlia* with *Oehliadiaphana* comb. nov. and an emended description of *Rhizoglomusvesiculiferum* comb. nov. in the Glomeromycotina. Nova Hedwigia 107(3–4): 501–518. 10.1127/nova_hedwigia/2018/0488

[B15] Błaszkowski J Niezgoda P Goto BT Kozłowska A (2018b) *Halonatospora* gen. nov. with *H.pansihalos* comb. nov. and *Glomusbareae* sp. nov. (Glomeromycota; Glomeraceae). Botany 96(11): 737–748. 10.1139/cjb-2018-0107

[B16] Błaszkowski J Ryszka P Kozłowska A (2018c) *Dominikialitorea*, a new species in the Glomeromycotina, and biogeographic distribution of *Dominikia.* Phytotaxa 338(3): 241–254. 10.11646/phytotaxa.338.3.2

[B17] Błaszkowski J Jobim K Niezgoda P Meller E Malinowski M Milczarski P Zubek S Magurno F Casieri L Bierza W Błaszkowski T Crossay T Goto BT (2021a) New glomeromycotan taxa, *Dominikiaglomerocarpica* sp. nov. and *Epigeocarpumcrypticum* gen. nov. et sp. nov. from Brazil, and *Silvaspora* gen. nov. from New Caledonia. Frontiers in Microbiology 12: 655910. 10.3389/fmicb.2021.655910 PMC8102679 33967994

[B18] Błaszkowski J Niezgoda P Meller E Milczarski P Zubek S Malicka M Uszok S Casieri L Goto BT Magurno F (2021b) New taxa in Glomeromycota: Polonosporaceae fam. nov., *Polonospora* gen. nov., and *P.polonica* comb. nov. Mycological Progress 20(8): 941–951. 10.1007/s11557-021-01726-4

[B19] Błaszkowski J Niezgoda P Zubek S Meller E Milczarski P Malicka M Goto BT Woźniak G Moreira H Magurno F (2021c) *Dominikiabonfanteae* and *Glomusatlanticum*, two new species in the Glomeraceae (phylum Glomeromycota) with molecular phylogenies reconstructed from two unlinked loci. Mycological Progress 20(2): 131–148. 10.1007/s11557-020-01659-4

[B20] Błaszkowski J Niezgoda P Zubek S Meller E Milczarski P Malinowski R Malicka M Uszok S Goto BT Bierza W Casieri L Magurno F (2022a) Three new species of arbuscular mycorrhizal fungi of the genus *Diversispora* from maritime dunes of Poland. Mycologia 114(2): 453–466. 10.1080/00275514.2022.2030081 35358026

[B21] Błaszkowski J Sánchez-García M Niezgoda P Zubek S Fernández F Vila A Al-Yahya’ei MN Symanczik S Milczarski P Malinowski R Cabello M Goto BT Casieri L Malicka M Bierza W Magurno F (2022b) A new order, Entrophosporales, and three new *Entrophospora* species in Glomeromycota. Frontiers in Microbiology 13: 962856. 10.3389/fmicb.2022.962856 PMC9835108 36643412

[B22] Błaszkowski J Yamato M Niezgoda P Zubek S Milczarski P Malinowski R Meller E Malicka M Goto BT Uszok S Casieri L Magurno F (2023) A new genus, *Complexispora*, with two new species, *C.multistratosa* and *C.mediterranea*, and *Epigeocarpumjaponicum* sp. nov. Mycological Progress 22(5): 34. 10.1007/s11557-023-01882-9

[B23] Błaszkowski J Zubek S Milczarski P Malinowski R Goto BT Niezgoda P (2024) *Glomusrugosae*, a new arbuscular mycorrhizal species in Glomeraceae (phylum Glomeromycota) from maritime sand dunes of Poland and an ash pond of Czech Republic. Phytotaxa 644(4): 271–280. 10.11646/phytotaxa.644.4.3

[B24] Chethana KWT Manawasinghe IS Hurdeal VG Bhunjun CS Appadoo MA Gentekaki E Raspé O Promputtha I Hyde KD (2021) What are fungal species and how to delineate them? Fungal Diversity 2021(109): 1–25. 10.1007/s13225-021-00483-9

[B25] Corazon-Guivin MA Cerna-Mendoza A Guerrero-Abad JC Vallejos-Tapullima A Carballar-Hernández S da Silva GA Oehl F (2019a) *Nanoglomusplukenetiae*, a new fungus from Peru, and a key to small-spored Glomeraceae species, including three new genera in the “*Dominikia* complex/clades”. Mycological Progress 18(12): 1395–1409. 10.1007/s11557-019-01522-1

[B26] Corazon-Guivin MA Cerna-Mendoza A Guerrero-Abad JC Vallejos-Tapullima A Carballar-Hernández S da Silva GA Oehl F (2019b) *Microkamienskia* gen. nov. and *Microkamienskiaperuviana*, a new arbuscular mycorrhizal fungus from Western Amazonia. Nova Hedwigia 109(3–4): 355–368. 10.1127/nova_hedwigia/2019/0551

[B27] Gomes SRBS de Queiroz MB Leroy JAS de Lima JLR Freire FADM Jobim K de Souza FA Goto BT (2022) Richness of Arbuscular Mycorrhizal Fungi in a Brazilian Tropical Shallow Lake: Assessing an Unexpected Assembly in the Aquatic-Terrestrial Gradient. Diversity 14(12): 1046. 10.3390/d14121046

[B28] Goto BT Maia LC (2006) Glomerospores: A new denomination for the spores of Glomeromycota, a group molecularly distinct from Zygomycota. Mycotaxon 96: 29–132.

[B29] Goto BT Queiroz MB Magurno F Souza FA Błaszkowski J (2024) How far have we progress in Glomeromycota taxonomy and systematic? IMS newsletter 5: 18–24.

[B30] Hall TA (1999) BioEdit: a user-friendly biological sequence alignment editor and analysis program for Windows 95/98/NT. Nuclei Acids Symposium Series 41: 95–98.

[B31] Katoh K Rozewicki J Yamada KD (2019) MAFFT online service: Multiple sequence alignment, interactive sequence choice and visualization. Briefings in Bioinformatics 20(4): 1160–1166. 10.1093/bib/bbx108 28968734 PMC6781576

[B32] Kornerup A Wanscher JH (1983) Methuen handbook of colour, 3^rd^ edn. Eyre Methuen, London, 252 pp.

[B33] Kozlov AM Darriba D Flouri T Morel B Stamatakis A (2019) RAxML-NG: A fast, scalable, and user-friendly tool for maximum likelihood phylogenetic inference. Bioinformatics 35(21): 4453–4455. 10.1093/bioinformatics/btz305 31070718 PMC6821337

[B34] Krüger M Stockinger H Krüger C Schüßler A (2009) DNA-based level detection of Glomeromycota: One PCR primer set for all arbuscular mycorrhizal fungi. The New Phytologist 183(1): 212–223. 10.1111/j.1469-8137.2009.02835.x 19368665

[B35] Miller MA Pfeiffer W Schwartz T (2010) Creating the CIPRES science gateway for inference of large phylogenetic trees. In: IEEE (Org.) Proceedings of the Gateway Computing Environments Workshop. 14 Nov 2010. IEEE, New Orleans, LA, 8 pp. 10.1109/GCE.2010.5676129

[B36] Morton JB Redecker D (2001) Two families of Glomales, Archaeosporaceae and Paraglomaceae, with two new genera *Archaeospora* and *Paraglomus*, based on concordant molecular and morphological characters. Mycologia 93(1): 181–195. 10.1080/00275514.2001.12063147

[B37] Niezgoda P Błaszkowski J Błaszkowski T Stanisławczyk A Zubek S Milczarski P Malinowski R Meller E Malicka M Goto BT Uszok S Casieri L Magurno F (2024) Three new species of arbuscular mycorrhizal fungi (Glomeromycota) and *Acaulosporagedanensis* revised. Frontiers in Microbiology 15: 1320014. 10.3389/fmicb.2024.1320014 PMC10896085 38410392

[B38] Oehl F Wiemken A Sieverding E (2003) *Glomusaureum*, a new sporocarpic arbuscular mycorrhizal fungal species from European grasslands. Journal of Applied Botany 77: 111–115.

[B39] Oehl F da Silva GA Goto BT Sieverding E (2011) Glomeromycota: Three new genera and glomoid species reorganized. Mycotaxon 116(1): 75–120. 10.5248/116.75

[B40] Oehl F Sánchez-Castro I Palenzuela J Silva G Sieverding E (2014) *Glomuscompressum*, a new arbuscular mycorrhizal fungus from different agro-ecosystems in Central Europe. Nova Hedwigia 99(3–4): 429–439. 10.1127/0029-5035/2014/0200

[B41] Oehl F Sánchez-Castro I Ferreira de Sousa NMF Silva G Palenzuela J (2015) *Dominikiabernensis*, a new arbuscular mycorrhizal fungus from a Swiss no-till farming site, and *D.aurea*, *D.compressa* and *D.indica*, three new combinations in *Dominikia.* Nova Hedwigia 101(1–2): 65–76. 10.1127/nova_hedwigia/2014/0235

[B42] Omar MB Bollan L Heather WA (1979) A permanent mounting medium for fungi. Bulletin British Mycological Society 13(1): 31–32. 10.1016/S0007-1528(79)80038-3

[B43] Queiroz MBD Jobim K Vista XM Leroy JAS Gomes SRBS Goto BT (2020) Occurrence of Glomeromycota species in aquatic habitats: A global overview. Mycotaxon 135(2): 469. 10.5248/135.469

[B44] Queiroz MB Leroy JAS Gomes SRBS Fiuza PO Goto BT (2022) Arbuscular Mycorrhizal Fungi (Glomeromycota) species inhabiting sediments of lentic and lotic Brazilian ecosystems: Addition of new global records for aquatic condition. Nova Hedwigia 115(1–2): 227–251. 10.1127/nova_hedwigia/2022/0701

[B45] Redecker D Schüßler A Stockinger H Stürmer SL Morton JB Walker C (2013) An evidence-based consensus for the classification of arbuscular mycorrhizal fungi (Glomeromycota). Mycorrhiza 23(7): 515–531. 10.1007/s00572-013-0486-y 23558516

[B46] Ronquist F Teslenko M van der Mark P Ayres DL Darling A Höhna S Larget B Liu L Suchard MA Huelsenbeck JP (2012) MrBayes 3.2: Efficient Bayesian phylogenetic inference and model choice across a large model space. Systematic Biology 61(3): 539–542. 10.1093/sysbio/sys029 22357727 PMC3329765

[B47] Sayre R Karagulle D Frye C Boucher T Wolff NH Breyer S Wright D Martin M Butler K Van Graafeiland K Touval J Sotomayor L McGowan J Game ET Possingham H (2020) An assessment of the representation of ecosystems in global protected areas using new maps of World Climate Regions and World Ecosystems. Global Ecology and Conservation 21: e00860. 10.1016/j.gecco.2019.e00860

[B48] Schüßler A Walker C (2010) The Glomeromycota. A Species List with New Families and New Genera. Gloucester: Royal Botanic Garden, Edinburgh.

[B49] Silva GAD, Assis DMDA, Sieverding E, Oehl F Four New Families of Arbuscular Mycorrhizal Fungi within the Order Glomerales. Preprints 2024, 2024081661. 10.20944/preprints202408.1661.v1

[B50] Smith SE Read DJ (2008) Mycorhizal symbiosis. 3^rd^ edn. Academic Press, Amsterdam, the Netherlands, 800 pp.

[B51] Stefanowicz AM Zubek S Stanek M Grześ IM Rożej-Pabijan E Błaszkowski J Woch MW (2019) Invasion of *Rosarugosa* induced changes in soil nutrients and microbial communities of coastal sand dunes. The Science of the Total Environment 677: 340–349. 10.1016/j.scitotenv.2019.04.408 31059877

[B52] Symanczik S Al-Yahya’ei MN Kozłowska A Ryszka P Błaszkowski J (2018) A new genus, *Desertispora*, and a new species, *Diversisporasabulosa*, in the family Diversisporaceae (order Diversisporales, subphylum Glomeromycotina). Mycological Progress 17(4): 437–449. 10.1007/s11557-017-1369-y

[B53] Tamura K Stecher G Peterson D Filipski A Kumar S (2013) MEGA6: Molecular Evolutionary Genetics Analysis Version 6.0. Molecular Biology and Evolution 30(12): 2725–2729. 10.1093/molbev/mst197 24132122 PMC3840312

[B54] Walker C (1983) Taxonomic concepts in the Endogonaceae: Spore wall characteristics in species descriptions. Mycotaxon 18: 443–455.

[B55] Wijayawardene NN Hyde KD Mikhailov KV Péter G Aptroot A Pires-Zottarelli CLA Goto BT Tokarev YS Haelewaters D Karunarathna SC Kirk PM Santiago ALCM de A Saxena RK Schoutteten N Wimalasena MK Aleoshin VV Al-Hatmi AMS Ariyawansa KGSU Assunção AR Bamunuarachchige TC Hans-Baral O Bhat DJ Błaszkowski J Boekhout T Boonyuen N Brysch-Herzberg M Cao B Cazabonne J Xue-Chen M Coleine C Dai D-Q Daniel H-M da Silva SBG de Souza FA Dolatabadi S Dubey MK Dutta AK Ediriweera A Egidi E Elshahed MS Fan X Felix JRB Galappaththi MCA Groenewald M Han L-S Huang B Hurdeal VG Ignatieva AN Jerônimo GH de Jesus AL Kondratyuk S Kumla J Kukwa M Li Q Lima JLR Liu X-Y Lu W Lumbsch HT Madrid H Magurno F Marson G McKenzie EHC Menkis A Mešić A Nascimento ECR Nassonova ES Nie Y Oliveira NVL Ossowska EA Pawłowska J Peintner U Pozdnyakov IR Premarathne BM AK Priyashantha H Quandt CA Queiroz MB Rajeshkumar KC Raza M Roy N Samarakoon MC Santos AA Santos LA Schumm F Selbmann L Selçuk F Simmons DR Simakova AV Smith MTh Sruthi OP Suwannarach N Tanaka K Tibpromma S Tomás EO Ulukapı M Vooren NV Wanasinghe DN Weber E Wu Q Yang EF Yoshioka R Youssef NH Zandijk A Gui-Zhang Q Zhang J-Y Zhao H Zhao R Zverkov OA Thines M Karpov SA (2024) Classes and phyla of the kingdom Fungi. Fungal Diversity. 10.1007/s13225-024-00540-z

[B56] Yu F Goto BT Magurno F Błaszkowski J Wang J Ma W Feng H Liu Y (2022) *Glomuschinense* and *Dominikiagansuensis*, two new Glomeraceae species of arbuscular mycorrhizal fungi from high altitude in the Tibetan Plateau. Mycological Progress 21(2): 32. 10.1007/s11557-022-01799-9

