# Corrigendum: Błaszkowski J, Zubek S, Milczarski P, Malinowski R, Niezgoda P, Goto BT (2025) New taxa and a combination in Glomerales (Glomeromycota, Glomeromycetes). MycoKeys 112: 253–276. https://doi.org/10.3897/mycokeys.112.136158

**DOI:** 10.3897/mycokeys.134.198490

**Published:** 2026-06-18

**Authors:** Janusz Błaszkowski, Szymon Zubek, Paweł Milczarski, Ryszard Malinowski, Piotr Niezgoda, Bruno Tomio Goto

**Affiliations:** 1 Department of Environmental Management, West Pomeranian University of Technology in Szczecin, Słowackiego 17, PL-71434 Szczecin, Poland Institute of Botany, Faculty of Biology, Jagiellonian University Kraków Poland; 2 Institute of Botany, Faculty of Biology, Jagiellonian University, 30-387, Kraków, Poland Departamento de Botânica e Zoologia, Universidade Federal do Rio Grande do Norte Natal Brazil; 3 Department of Genetic, Plant Breeding & Biotechnology, West Pomeranian University of Technology in Szczecin, Słowackiego 17, PL-71434 Szczecin, Poland Department of Environmental Management, West Pomeranian University of Technology in Szczecin Szczecin Poland; 4 Departamento de Botânica e Zoologia, Universidade Federal do Rio Grande do Norte, Campus Universitário, 59078–900, Natal, RN, Brazil Department of Genetic, Plant Breeding & Biotechnology, West Pomeranian University of Technology in Szczecin Szczecin Poland

We have been informed that the names *Delicatispora* and *Delicatispora indica* are not validly published. A full and direct reference to the basionym *Glomus indicum* was omitted which rendered the name of the type species of the new genus invalid and also that of the generic name *Delicatispora* (Madrid code: Art. 40.1, see Arts 40.3 and Arts 6.3, 12.1 and Art. 41.5; Turland et al. 2025). The names are validated herein.

## Delicatispora

Taxon classification

Fungi

GlomeralesDominikiaceae

Błaszk., Niezgoda & B.T. Goto gen. nov.

25D2120D-F71A-5A08-B7F9-44A67B2AC38A

863205

### Note.

For a detailed description see Błaszkowski et al., MycoKeys 112: 261 (2025).

### Type species.

*Delicatispora indica* (Błaszk., Wubet & Harikumar), Błaszk., Niezgoda & B.T. Goto, comb. nov. MB 863206.

### Basionym.

*Glomus indicum* Błaszk., Wubet & Harikumar, Botany (Ottawa) 88: 134 (2010).

## Supplementary Material

XML Treatment for Delicatispora

## Additional information

### Artificial Intelligence (AI) use

The authors accept full responsibility for the content of the manuscript, including the disclosure of any use of AI.

No AI tools were used in the preparation of this manuscript.

### Author ORCIDs

J. Błaszkowski https://orcid.org/0000-0003-3688-164X

S. Zubek https://orcid.org/0000-0003-1338-9572

P. Milczarski https://orcid.org/0000-0003-2622-8938

R. Malinowski https://orcid.org/0000-0003-3413-2653

P. Niezgoda https://orcid.org/0000-0002-0560-6154

B.T. Goto https://orcid.org/0000-0001-6157-4954

### Data availability

All of the data that support the findings of this study are available in the main text.
