# Supplementary figures and images for: ﻿New taxa and a combination in Glomerales (Glomeromycota, Glomeromycetes)
Source: MycoKeys. 2025 Jan 22;112:253–76. doi: 10.3897/mycokeys.112.136158 (PMC11780324; doi:10.3897/mycokeys.112.136158)

Fig. S1, cont.

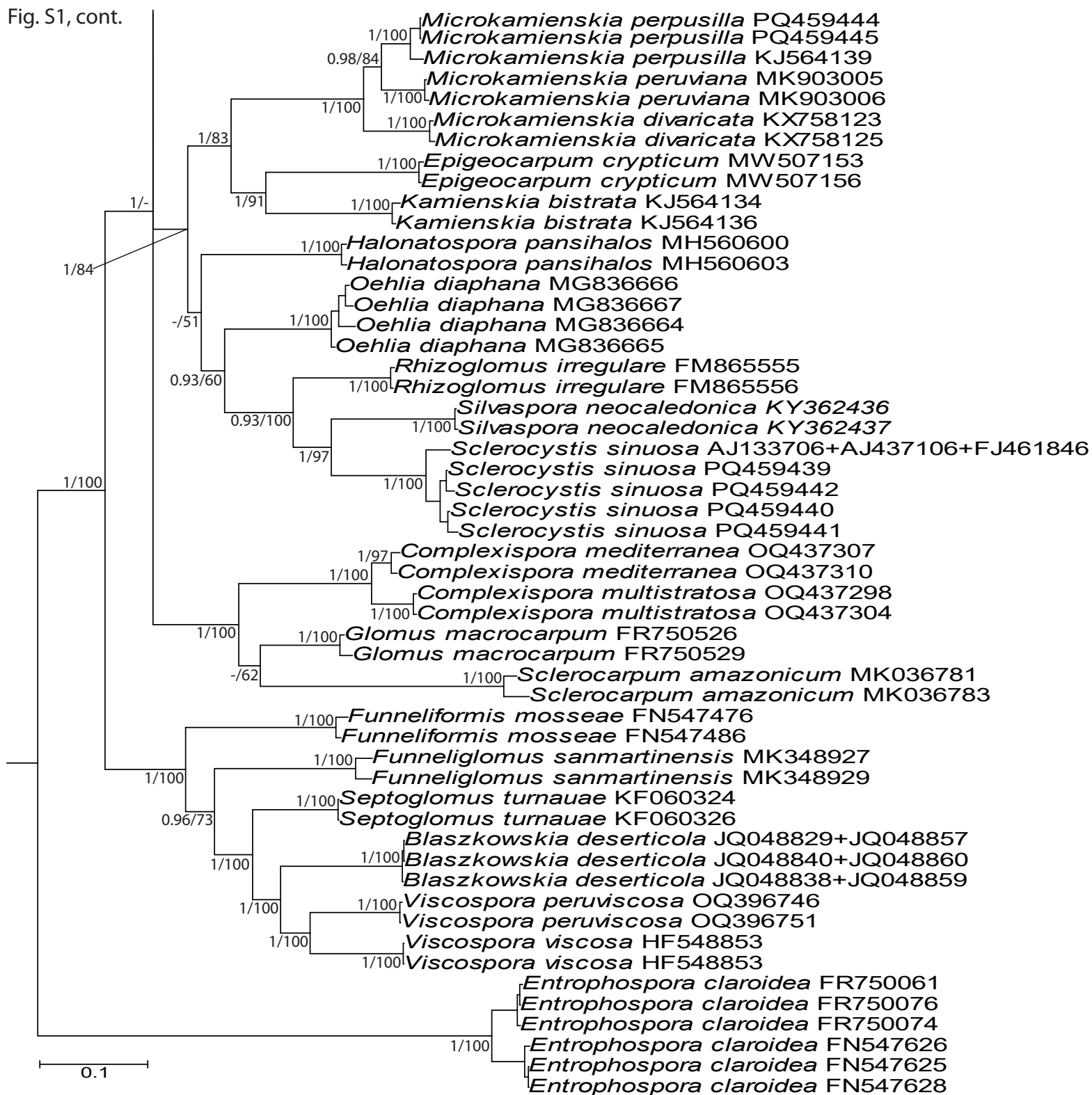

Supplement: Supplementary material 1 — 50% majority-rule consensus tree from the Bayesian analysis of sequences of 45S nuc rDNA sequences of Macrodominikiacompressa, Delicatisporaindica, Dominikiaparaminuta, 33 other species of Glomerales, as well as Entrophosporaclaroidea serving as outgroup [file mycokeys-112-253-s001.zip › 136158_1C-1-A_revised_Supplementary_material_1,_continued.pdf]

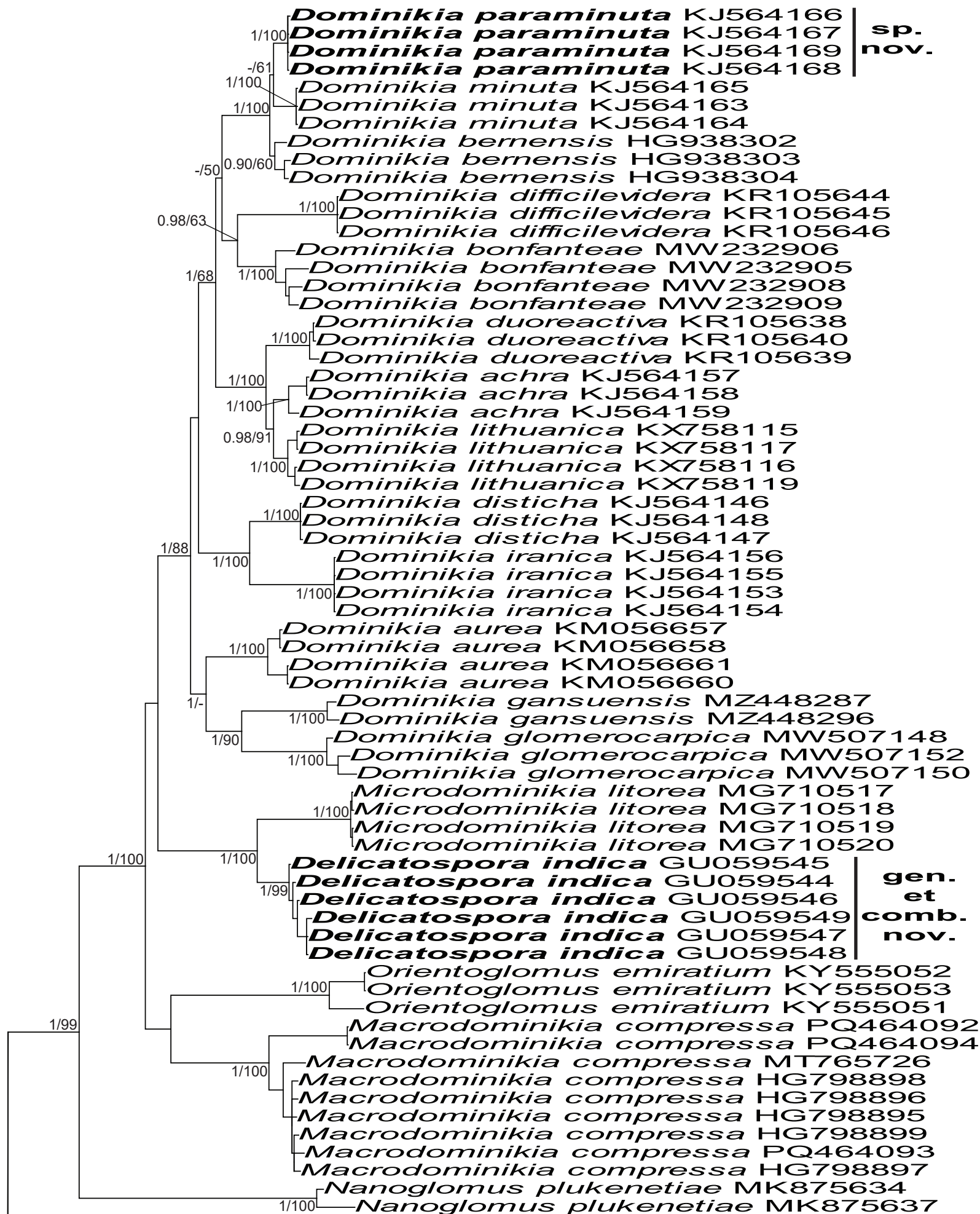

Supplement: Supplementary material 1 — 50% majority-rule consensus tree from the Bayesian analysis of sequences of 45S nuc rDNA sequences of Macrodominikiacompressa, Delicatisporaindica, Dominikiaparaminuta, 33 other species of Glomerales, as well as Entrophosporaclaroidea serving as outgroup [file mycokeys-112-253-s001.zip › 136158_1C-1-A_revised_Supplementary_material_1.pdf]

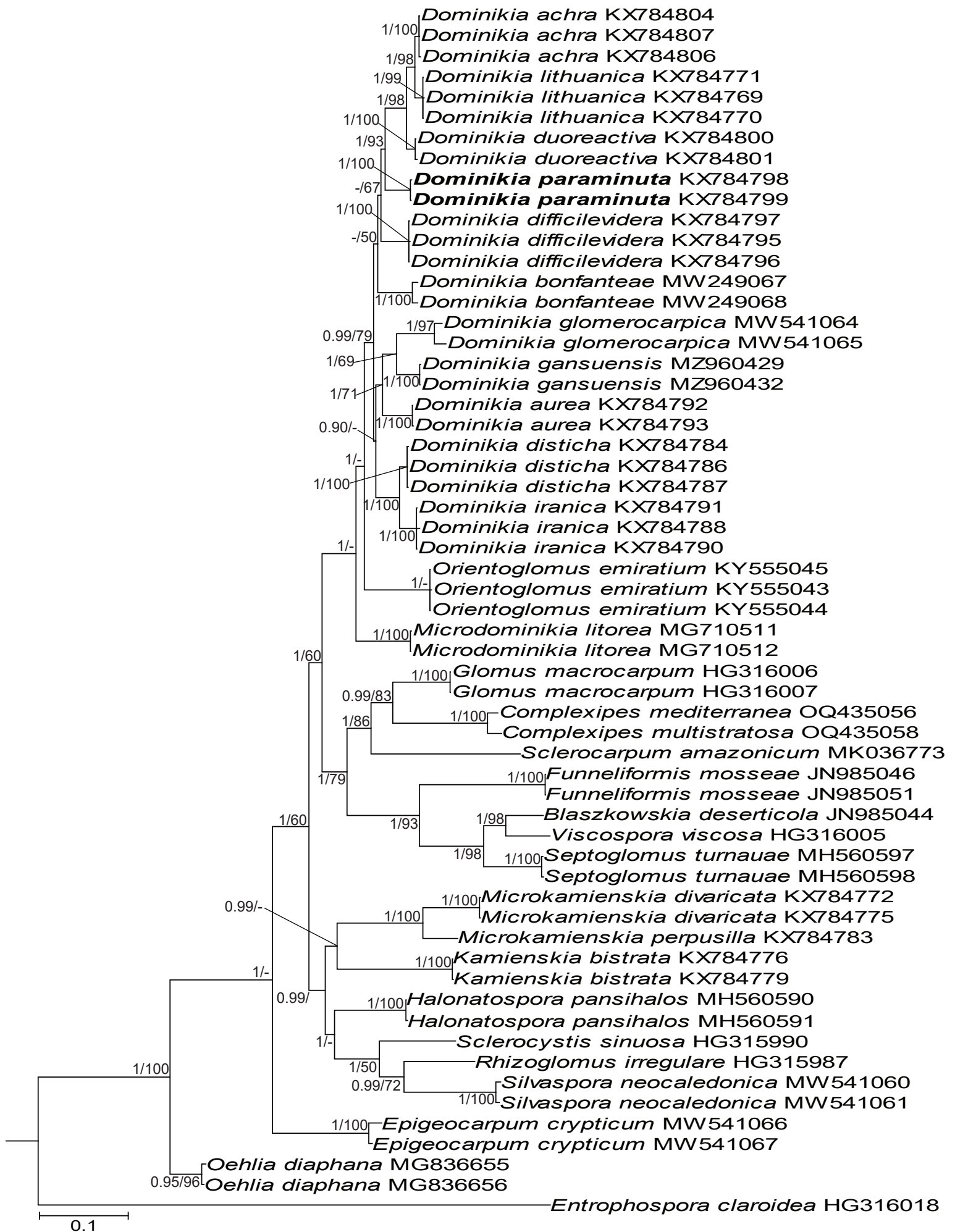

Supplement: Supplementary material 2 — 50% majority-rule consensus tree from the Bayesian analysis of sequences of rpb1 sequences of Dominikiaparaminuta, 29 other species of Glomerales, as well as Entrophosporaclaroidea serving as outgroup [file mycokeys-112-253-s002.pdf]
